# Sleep Timing in Patients with Precocious and Delayed Pubertal Development

**DOI:** 10.3390/clockssleep1010013

**Published:** 2019-02-16

**Authors:** Elena Jessen, Celine Vetter, Till Roenneberg, Klaus-Peter Liesenkötter, Helene Werner, Oskar G. Jenni, Erwin Lankes, Oliver Blankenstein, Uta Neumann, Birgit Köhler, Susanna Wiegand, Heiko Krude, Peter Kühnen

**Affiliations:** 1 Institute for Experimental Pediatric Endocrinology, Charité—Universitätsmedizin Berlin, corporate member of Freie Universität Berlin, Humboldt-Universität zu Berlin, and Berlin Institute of Health, 13353 Berlin, Germany; 2 Department of Integrative Physiology, University of Colorado at Boulder, Boulder, CO 80309, USA; 3 Institute of Medical Psychology, Ludwig-Maximilians-University, 80336 Munich, Germany; 4 Endokrinologikum, 10117 Berlin, Germany; 5 Child Development Center, University Children’s Hospital Zurich, 8050 Zurich, Switzerland; 6 Center for Chronic Sick Children (SPZ), Charité—Universitätsmedizin Berlin, Corporate Member of Freie Universität Berlin, Humboldt-Universität zu Berlin, and Berlin Institute of Health, 13353 Berlin, Germany; 7 Department for Pediatric Endocrinology and Diabetes, Charité—Universitätsmedizin Berlin, Corporate Member of Freie Universität Berlin, Humboldt-Universität zu Berlin, and Berlin Institute of Health, 13353 Berlin, Germany

**Keywords:** chronotype, puberty, circadian clock, sleep, adolescence

## Abstract

Previous studies have reported a shift in the timing of sleep during adolescence toward a later time. To date, it is unclear whether hormonal changes during puberty might contribute to this change in sleeping behavior. We systematically assessed pubertal development and sleep timing in a cross-sectional case-control study in girls with precocious (*n* = 42) and boys with delayed pubertal development (*n* = 19). We used the Munich ChronoType Questionnaire and the Children’s ChronoType Questionnaire to assess sleep timing in patients and age- and sex-matched controls (*n* = 309) and used the midpoint of sleep on free days, corrected for potential sleep debt accumulated during the school week, as a marker for sleep timing. Compared to the controls, girls with central precocious puberty showed a delay in sleep timing of 54 min, and girls with premature pubarche slept on average 30 min later. Male adolescents with delayed pubertal development showed an average sleep midpoint that was 40 min earlier compared to the control group. The results of this pilot study suggest an association between pubertal onset and shifts in sleep timing, which is a novel finding in human sleep behavior. Prospective studies in larger cohorts will be needed to examine the robustness and generalizability of the findings.

## 1. Introduction

Sleep timing, a key aspect of sleep behavior, varies largely within the population [1]. Sleep timing emerges from the interplay between the circadian system and sleep homeostasis and differs between individuals who wake up early in the morning and individuals who wake up late in the day. This variation in sleep timing has been used as a proxy for chronotype, or the individual phase of entrainment [2,3,4,5,6,7]. Recently, studies have reported a biphasic shift of sleep timing in parallel with chronological maturation [1,8,9,10,11], with adolescents continuously shifting later during adolescence, and then becoming earlier again in early adulthood [1,12,13]. Adolescents on average have a later phase of entrainment and this is paralleled by their sleep timings, especially on non-school days when sleep timing is not affected by school start times, but at least in part regulated by the circadian system. This phase delay leads to a mismatch with early school and work start times, a phenomenon also referred to as social jetlag [14]. In teenagers, who have overall later chronotypes than adults and therefore are more likely to experience this mismatch even with daytime schedules, social jetlag has been associated with worse academic achievements. In adults, higher levels of social jetlag have been associated with adverse metabolic profiles and obesity [12,15,16,17,18,19,20,21].

Late adolescent sleep timing has been attributed to environmental factors [22,23,24,25], but might also be related to endogenous hormonal changes during puberty [26,27]. This hypothesis is supported by rodent studies where gonadectomized rodents showed a shift toward later timings in their day–night activity rhythm [28,29,30,31]. We therefore examined the association between pubertal development and sleep timing in children with precocious or delayed puberty using validated questionnaires [1,32] and compared those to age- and sex-matched controls. Pubertal development is divided into pubarche (occurrence of, e.g., pubic hair), which is dependent on adrenal gland function, and central puberty (leading to breast development, menarche), which is related to the secretion of gonadotropins by the hypothalamus and pituitary and estrogen/testosterone production by the ovaries or testis. Premature thelarche indicates transient breast development without stimulation by gonadotropins. We hypothesized that the precocious onset of pubertal development (i.e., premature pubarche and precocious central puberty) would be associated with a later sleep timing, while delayed pubertal development would be associated with earlier sleep timing phenotypes.

## 2. Results 

### 2.1. Sleep Timing in Patients with Precocious or Delayed Pubertal Development

**Figure 1:**
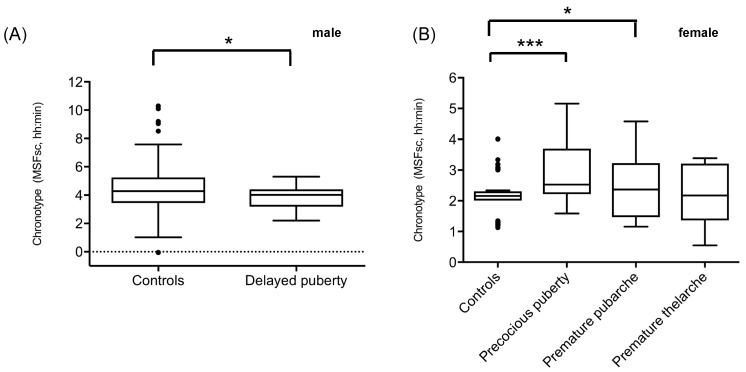
Chronotype of patients with delayed or early pubertal development compared to control individuals. (**A**) MSFsc (corrected midpoint of sleep on free days) was significantly earlier in the boys with delayed pubertal development compared to a healthy control group. (**B**) The MSFsc was significantly shifted toward a later time in girls with early onset of puberty (precocious puberty and premature pubarche) compared to a healthy control group. No differences of chronotype were identified in a group of girls with premature thelarche in relation to the control group. In the box plots, the horizontal line represents the median and the bars indicate the 5th and the 95th percentiles. The MSFsc is documented in decimal, military time. * *p* < 0.05; *** *p* < 0.001

**Figure 2:**
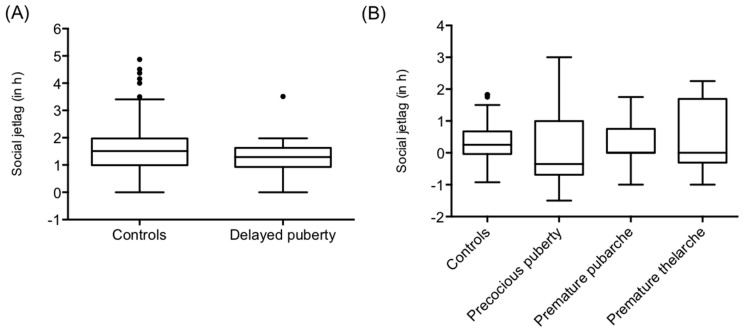
Social jetlag. (**A**) We did not observe systematic differences between boys with delayed pubertal development and the control group (**B**) Furthermore the social jetlag did not differ between girls with precocious puberty, premature pubarche, or premature thelarche compared to age-matched controls. In the box plots, the horizontal line represents the median and the bars indicate the 5th and the 95th percentiles.

The characteristics of all cohorts are summarized in Table 1. We observed that boys with a delayed pubertal development had a significantly earlier chronotype than the male database control cohort of the same age (*n* = 19) (Figure 1A; Table 2). Girls with precocious central puberty (*n* = 13) and premature pubarche (*n* = 19), in turn, showed significantly later sleep timing as compared to their age-matched female controls (Figure 1B; Table 2). However, sleep timing did not differ between the 10 girls with a premature thelarche and the controls (Figure 1B, Table 2). However, the database control group was slightly younger than the patient cohort with precocious puberty. Social jetlag did not differ between controls and boys with delayed puberty (Figure 2A; Table 2) or girls with an early pubertal development (Figure 2B; Table 2).

In secondary analyses, we examined potential differences in sleep duration. Boys with delayed pubertal development slept significantly longer on school days compared to controls (Supplemental Figure S1A; Table 2), while sleep duration on free days was comparable across both groups (Supplemental Figure S1B; Table 2). We observed no differences in the duration of sleep on work and on free days in girls compared to the female controls (Supplemental Figure S1C,D; Table 2). 

### 2.2. Hormone Levels in Patients with Abnormal Pubertal Development

**Figure 3:**
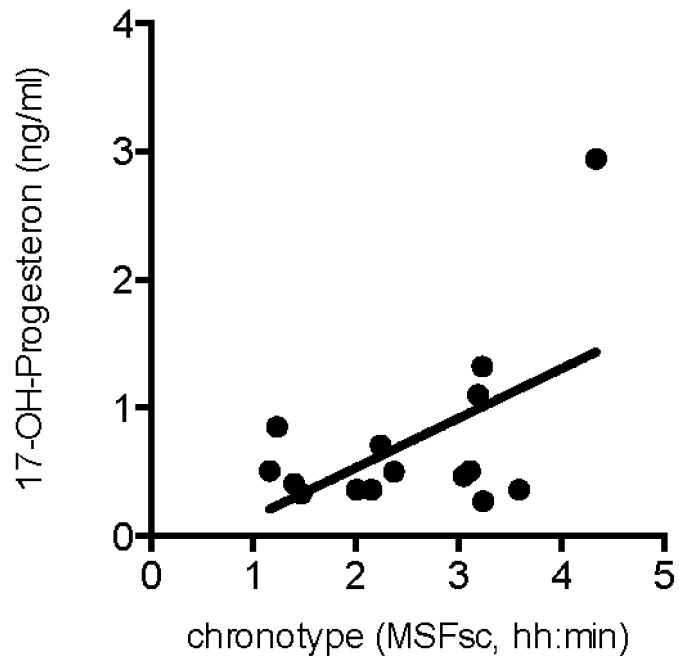
17-hydroxy-progesterone levels. Patient blood values were available only for a subgroup of participants (*n* = 15). A significant positive correlation between the 17-OHP levels of patients with premature pubarche and sleep timing (assessed by MSFsc) was observed within this small cohort.

Plasma hormone levels were available for 12 patients with delayed puberty, 8 patients with precocious puberty, 15 patients with premature pubarche, and 3 patients with premature thelarche. We observed a positive correlation between sleep timing and the levels of the adrenal androgen-precursor steroid 17-OHP (*r* = 0.54, *p* = 0.036, false discovery rate (FDR) corrected *p* = 0.1105; Figure 3) in girls with premature pubarche. Blood parameters were available for 8 girls with precocious puberty; however, in this limited sample size no other association with hormonal parameters reached statistical significance after outlier correction (>2 SD from mean).

No further information about pulsatile gonadotropin secretion was available at this stage. All correlation coefficients between sleep timing and LH (luteinizing hormone), stimulated FSH (follicle stimulating hormone), testosterone, estradiol, 17-OHP, or DHEAS were below 0.2. Correction for multiple comparisons was performed by using FDR, giving a corrected level of significance alpha threshold of 0.16. 

## 3. Discussion

Several studies have reported a shift in sleep timing during adolescence that, on average, has a magnitude of about 60–120 min [13]. Although sleep timing is variable among individuals, this trend has been described in several populations [1,8,9,11,33] and replicated in a longitudinal study [34]. While several studies in rodents point toward an impact of pubertal hormones on chronotype shift [35], human evidence for such a link is scarce. We examined patients with the rare condition of precocious (incidence: 1:5000–1:10,000) or delayed puberty onset. To explore a potential role of hormone levels in this phenomenon, we performed a cross-sectional pilot study in these patients and collected sleep information using established, validated questionnaires [32,36]. Children and adolescents with abnormal pubertal development differed from their age- and sex-matched controls, in the hypothesized directions. Interestingly, only girls with accelerated puberty and with substantial clinical symptoms of precocious puberty or premature adrenarche showed later sleep timing (54 min and 30 min, respectively) as compared to controls. Girls with milder forms of premature thelarche—without a central pubertal stimulation—did not differ from controls. Inversely, boys with a delayed puberty slept on average 40 min earlier. Together, these data imply that human pubertal development might be linked with sleep timing. It is noteworthy though, that the sample size was small and that associations did not survive multiple testing adjustments. Therefore, prospective replication in larger settings is warranted. Furthermore, girls are entering puberty at a younger age than boys for unknown reasons. This sexual dimorphism affects sleep timing and chronotype, leading to differences between boys and girls. These differences are additionally observed before and after puberty [1,32].

Plasma hormone levels were available in a subset of individuals with altered pubertal development. We observed a positive correlation between the 17-OHP levels and the sleep timing in girls with premature pubarche. These results support the notion that pubertal development is linked to sleep phenotypes, as the elevation of these hormone levels represents the activation of pubarche [26,37]. Recently, Foley et al. reported a prospective association between trajectories in pubertal timing and sleep behavior in a large US cohort [31]; those results are in line with our observation that higher 17-OHP levels were associated with a later sleep timing in our study. We did not identify further associations between plasma hormone levels and sleep timing. Because of the limited sample size, we further emphasize the need of replication efforts examining sleep behavior and pubertal stages. 

Boys with delayed puberty slept longer on work/school days compared to controls. This could be due to the differences in sleep timing, as boys with delayed pubertal development had earlier sleep onsets on average than the database control group. Given that earlier sleep timing is in better alignment with early school start times—in Germany, schools typically start at around 8:00 a.m.—those boys experienced less sleep curtailment than the controls of the database control cohort. While controls showed nearly a 1.1 h discrepancy between sleep duration on school and free days, boys with delayed puberty only had a 0.3 h difference. This difference was not statistically significant, but was in line with previous reports of increasing sleep debt during the week as a function of sleep timing [12]. 

This is an exploratory study with a limited number of patients. However, the magnitude of the differences that were found in the hypothesized directions suggests a strong biological effect that needs to be replicated in a larger cohort of children. Our study has several further limitations. First, the cross-sectional design does not allow the disentanglement of the directionality of associations. Ideally, a prospective study [34], would assess environmental factors, individual circadian phase (for example, by means of dim-light melatonin onset [38]), objective sleep behavior, as well as all established clinical and objective hormonal markers of pubertal development, and genetic information. Second, due to limited access to control data, the database control group was slightly younger than the group of patients with central precocious puberty. Furthermore, no information about pubertal stages or any hormonal levels was available for this database control cohort, and this hampered direct comparisons of sleep behavior by pubertal stages. Precocious and delayed pubertal development are rare clinical conditions though, and it is unlikely that either database comprised a substantial number of individuals who were precocious or delayed in their pubertal development. If exposure misclassification occurred, it was likely non-differential, which would result in an underestimation of the association. Another limitation relates to the question of which physiological component of pubertal regulation is leading to changes in sleep timing. The onset of puberty is reflected by the release of hypothalamic peptides that will in turn trigger the pituitary gonadotropins FSH and LH, which then stimulate the gonadal secretion of sex steroids. Those sex steroids (i.e., estrogen and testosterone) finally lead to the visible clinical signs of pubertal development. In addition, the adrenal release of androgens leads to pubic hair development. Several rodent studies have shown that both, the adrenal and the central hypothalamic–pituitary axis can have an impact on circadian clock output [39,40,41], and that androgens and estrogens can modify clock-gene expression [42,43]. Moreover, gonadectomized animals do not show a physiological activity change during adolescence, a phenomenon that can be restored when these animals are treated with testosterone or estrogen [44,45]. While a direct translation of such findings is not appropriate, given the fundamental differences in the length of sexual maturation, timing of activity, and exposure to social and environmental factors, those results largely suggest a potential role for hormonal pathways related to changes in behavioral timing not only in rodents but also in humans. 

## 4. Material and Methods

This study has been approved by the ethical committee of the Charité Universitätsmedizin Berlin (No. EA2/032/12) and the ethical committee of the Ludwig Maximilian University. The patients and their legal guardians gave written informed consent, and the study was conducted in accordance with the Declaration of Helsinki.

### 4.1. Patient Cohort

All patients were recruited in the outpatient clinic of the Department of Pediatric Endocrinology (Charité Universitätsmedizin Berlin) and the outpatient clinic of the Endokrinologikum (Dr. K.P. Liesenkötter, Berlin, Germany). We only included children in the study if their diagnostic workup revealed an idiopathic precocious or delayed pubertal development, excluding other primary causes such as adrenal, pituitary, or gonadal tumors; syndromic cases; or patients with congenital adrenal hyperplasia. The definitions for precocious and delayed pubertal development were based on international guidelines [46]. A clinical Tanner stage of ≥2 defines an onset of puberty. Tanner stages of the breast (girls), genitalia (boys), and pubic hair (both) were recorded. We also included girls with premature thelarche (clinically defined by transient breast development without the stimulation of gonadotropins before the age of 8) and girls with premature adrenarche (clinically defined by the occurrence of pubic hair without stimulation of gonadotropins before the age of 8). Delayed pubertal development was defined as an absence of pubertal clinical findings (Tanner stage of 1) at an age above 13 years in girls and 14 years in boys, respectively [37]. In total, 74 patients were recruited with these disturbed pubertal developments. Because of the low number of males in the precocious puberty group (*n* = 10) and the low numbers of females in the cohort with delayed puberty (*n* = 3), we only included boys with delayed and girls with precocious puberty a posteriori in the analysis (finally 61 out of the initial 74 patients were included in the analysis). For this reason, 19 patients with delayed puberty, 13 individuals with precocious central puberty, 19 patients with premature pubarche, and 10 individuals with premature thelarche were included within this proof-of-concept study (Table 1).

### 4.2. Database Control Cohorts

The study included two different control groups, depending on their age. We compared patients with delayed puberty to a convenience sample of age- and sex-matched controls (*n* = 240; age: 15.1 ± 1.2 years) from the Munich ChronoType Questionnaire (MCTQ) database (www.theWeP.org), while younger children (<10 years) with precocious puberty were compared to age- and sex-matched controls from the Children’s ChronoType Questionnaire (CCTQ) database (*n* = 69; age: 6.4 ± 1.2 years). The latter database control group was slightly younger than the precocious puberty group. Information about pubertal stages or hormonal parameters of the participants was not available. 

### 4.3. Sleep Assessments

The chronotype assessment in patients older than 10 years was performed with the Munich ChronoType Questionnaire (MCTQ) and in patients younger than 10 years with the Children’s ChronoType Questionnaire (CCTQ) [1,32,36], both of which use the midpoint of sleep on free days corrected for the sleep debt accumulated over the workweek (MSFsc; corrected midpoint of sleep on free days) as a surrogate for chronotype. Social jetlag was calculated as the absolute difference between the midpoint of sleep on free days and on work days [14]. Both questionnaires also allow the computation of average sleep duration on free and work days. The use of two chronotype questionnaires was necessary, because only the CCTQ is established and evaluated in children below 10 years of age. However, the CCTQ is based on the MCTQ. Both questionnaires are well established and have been validated to quantify sleep behavior and sleep. 

### 4.4. Laboratory Measurements

Puberty-associated hormones such as the gonadotropins LH and FSH (basal and GnRH (gonadotropin releasing hormone) stimulated), as well as the sex steroid hormones testosterone, estradiol, 17-OHP (17-hydroxyprogesterone), and DHEAS (dehydroepiandrosteronesulfate) were measured at the Labor Berlin-Charité Vivantes GmbH according to standard protocols. Blood collection was performed during the first presentation of the patient within the outpatient clinic between 8:30 and 9:00 a.m. The chronotype questionnaire and blood collection were performed on the same day. 

### 4.5. Statistical Analysis

Statistical analyses were performed using SPSS (SPSS Inc., Chicago, IL, USA, version 23.0). We examined whether patients with precocious puberty had later sleep timings as compared to their matched controls using a one-sided t-test. In addition, we also examined whether patients with delayed pubertal development showed earlier sleep timings as compared to controls. In the secondary analysis, we examined the association between pubertal stage and sleep duration, expecting that children with precocious puberty would sleep less than controls, and that individuals with delayed pubertal development would sleep longer [47]. Outliers were defined by ±2 standard deviations, and we tested the robustness of the results in a sensitivity analysis excluding potential outliers, whenever appropriate. 

We report results of this pilot study with a significance level of alpha set to 0.05. Finally, in exploratory analyses, we examined whether the hormonal markers of pubertal development were higher in patients with later sleep timings. In case the data were not normally distributed, as tested with the Shapiro–Wilk test, we used adequate non-parametric tests (Spearman correlation) to examine the robustness of the aforementioned associations. We also report *p*-values adjusted for multiple testing by implementing a false discovery rate (FDR) [48].

## 5. Conclusions

Taken together, we observed sleep timing differences in patients with precocious or delayed pubertal development compared to control database groups. This support the notion that endogenous factors might contribute to sleep timing during puberty. This deserves further attention, and future studies are needed examining robustness of our results, potential interactions with environmental factors, and generalizability across healthy and patient populations. 

## Figures and Tables

**Table 1:** Sample characteristics for patients (by puberty status) and a database control sample. Pubertal development was assessed using Tanner stages. A clinical Tanner stage of ≥2 defines an onset of puberty and Tanner stage of 5 represents the mature adult clinical status. For the database control cohorts, no information about pubertal stages was available.

	Delayed Puberty	Precocious Central Puberty	Premature Pubarche	Premature Thelarche	Database Control Group, Adolescents (Roenneberg et al.)	Database Control Group, Children (Werner et al.)
Samples size	19 boys	13 girls	19 girls	10 girls	240	69
Age (years)	15.7 ± 1.1	8.0 ± 1.3	6.6 ± 1.3	5 ± 2.6	15.1 ± 1.2	6.4 ± 1.2
Sex (female/male)	0/19	13/0	19/0	10/0	0/240	69/0
Tanner stage (breast)	n/a	2.9 ± 0.9	1.3 ± 0.8	2.2 ± 0.4	n/a	n/a
Tanner stage (genitalia)	2.0 ± 1	n/a	n/a	n/a	n/a	n/a
Tanner stage (pubic hair)	2.0 ± 1	2.5 ± 1.3	2.2 ± 0.9	1.0 ± 0	n/a	n/a

**Table 2:** Chronotype, sleep, and social jetlag by patient and database control sample. All patient groups were analyzed with respect to chronotype, sleep duration, and social jetlag (mean value ± SD) and these data were compared to the results of control-database groups. *P*-values, false discovery rate (FDR) corrected *p*-values, and effect size (Cohen’s *d*) are noted within the field.

	Delayed Puberty	Precocious Central Puberty	Premature Pubarche	Premature Thelarche	Database Control Group, Adolescents (Roenneberg et al.)	Database Control Group, Children (Werner et al.)	Statistical Test
Chronotype (MSFsc)	3.8 ± 0.8 *p* = 0.039 FDR cor. *p* = 0.1248 *d* = 0.23	3.0 ± 1.1 *p* < 0.001 FDR cor. *p* = 0.008 *d* = 0.34	2.58 ± 1.0 *p* = 0.03 FDR cor. *p* = 0.1248 *d* = 0.24	2.2 ± 1.0 *p* = 0.7314 FDR cor. *p* = 0.7802	4.46 ± 1.4	2.1 ± 0.6	- T-test (one sided) - Effect size (Cohen’s *d*)
Sleep duration on school days (h)	8.0 ± 1.5 *p* < 0.001 FDR cor. *p* = 0.008 *d* = 0.55	10.1 ± 0.8 *p* = 0.2088 FDR cor. *p* = 0.4176	10.04 ± 0.7 *p* = 0.0643 FDR cor. *p* = 0.1715	10.12 ± 1.0 *p* = 0.3144 FDR cor. *p* = 0.4895	7.3 ± 1.0	10.4 ± 0.6	- T-test (one sided) - Effect size (Cohen’s *d*)
Sleep duration on free days (h)	8.3 ± 1.5 *p* = 0.47 FDR cor. *p* = 0.5785	10.19 ± 1.0 *p* = 0.052 FDR cor. *p* = 0.1248	10.43 ± 0.9 *p* = 0.2014 FDR cor. *p* = 0.4176	10.65 ± 1.6 *p* = 0.9191 FDR cor. *p* = 0.9191	8.4 ± 1.3	10.68 ± 0.7	- T-test (one sided)
Social jetlag (h)	1.32 ± 0.74 *p* = 0.3255 FDR cor. *p* = 0.4895	0.09 ± 1.28 *p* = 0.3365 FDR cor. *p* = 0.4895	0.39 ± 0.74 *p* = 0.6642 FDR cor. *p* = 0.7591	0.52 ± 1.15 *p* = 0.3792 FDR cor. *p* = 0.5056	1.52 ± 0.87	0.318 ± 0.61	- T-test (one sided)

## Supplementary Materials

Supplementary materials can be found at https://www.mdpi.com/2624-5175/1/1/13/s1.
