# Supplementary material for: Sleep Timing in Patients with Precocious and Delayed Pubertal Development
Source: Clocks Sleep. 2019 Feb 16;1(1):140–50. doi: 10.3390/clockssleep1010013 (PMC7509672; doi:10.3390/clockssleep1010013)
Supplement: Supplementary file 1 [file clockssleep-01-00013-s001.pdf]

Sleep Timing in Patients with Precocious and Delayed Pubertal Development

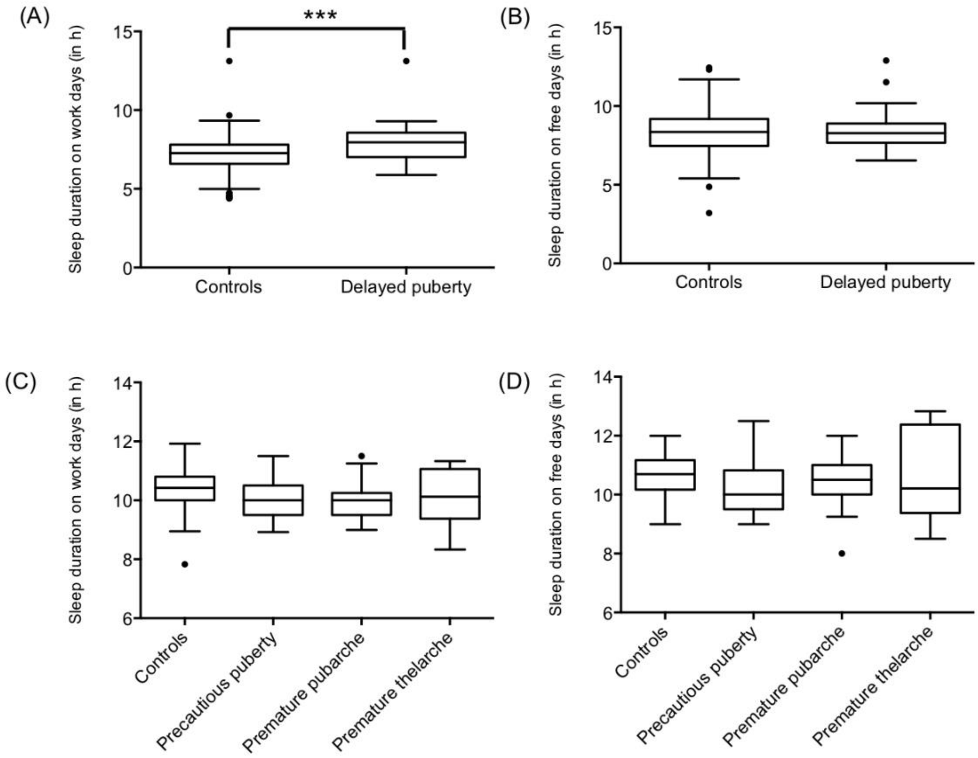

Figure S1. Analysis of sleep duration. (**A**) The sleep duration on work days was significantly longer in the group of boys with delayed pubertal development compared to the healthy control group. (**B**) Sleep duration on free days did not differ in boys with a delayed pubertal development. (**C**,**D**) No differences were observed in the sleep duration either on work or on free days in girls with early puberty compared to the control group. In the box plots, the horizontal line represents the median and the bars indicate the 5th and the 95th percentiles. *** $p < 0.001$
